# Zika: How safe is India?

**DOI:** 10.1186/s40249-016-0234-6

**Published:** 2017-01-31

**Authors:** C. George Priya Doss, R. Siva, B. Prabhu Christopher, Chiranjib Chakraborty, Hailong Zhu

**Affiliations:** 1 School of Biosciences and Technology, VIT University, Vellore, Tamil Nadu 632014 India; 2 VIT-BS, VIT University, Vellore, Tamil Nadu 632014 India; 3 Department of Bioinformatics, School of Computer and Information Sciences, Galgotias University, Greater Noida, Uttar Pradesh 201310 India; 4 Integrated Bioinformedicine and Translational Science, School of Chinese Medicine, Hong Kong Baptist University, 7 Baptist University Road, Hong Kong, China

**Keywords:** Zika virus, Preventive measures, India

## Abstract

Zika virus, which originated from a forest in Uganda, has affected countries in Africa, Latin America and Asia. Most people infected with Zika are asymptomatic and present with clinical manifestations ranging from mild fever to severe neurological disorders. Recent outbreaks in Southeast Asian countries, Centers for Disease Control and Prevention has warned pregnant woman to avoid nonessential traveling to 11 Asian countries. Reports about the sexual transmission route of Zika have pushed the World Health Organization to declare it a ‘public health emergency’. Having this current warning status, it has become mandatory to consider where second highly populated country India stands in terms of spreading awareness and taking precautionary measures against the Zika virus infection. Therefore, this paper aims to highlight the importance of Zika in Indian population by considering several indicators such as the population size and ratio, rates of mortality, closely related diseases, government initiatives, and other micro-level factors which are prone to Zika effects.

**Electronic supplementary material:**

The online version of this article (doi:10.1186/s40249-016-0234-6) contains supplementary material, which is available to authorized users.

## Multilingual abstract

Please see Additional file 1 for translation of the abstract into the five official working languages of the United Nations.

## Background

Zika virus (ZIKV) originated from Uganda’s Zika forest in late 1940’s has conquered global attention due to its virulent nature. Though Zika had its origin in African forest, the disease now affects around 70 countries of the South Americas, Pacific Islands,and Souhtheast Asia (WHO Situation Report, 16th October, 2016). For the past seven decades, researchers have tracked the route path of mosquito-borne ZIKV from the rhesus monkey in Uganda to humans in Latin America and Caribbean countries, with Anguilla being the latest country affected.

ZIKV is primarily transmitted to people through the bite of female *Aedes* mosquitoes, including *A. aegypti* and *A. albopictus*, which are also responsible for spreading chikungunya virus (CHIKV), yellow fever, and dengue (DENV) [1]. The Center for Disease Control and Prevention U.S (CDC) has reported that 80% of the cases infected outside Africa with ZIKV are asymptomatic and associated with an illness characterized by skin rashes, joint pain, low-grade fever, conjunctivitis, severe neurological disorders, microcephaly, and Guillain-Barré syndrome [2].

Various recent studies have reviewed the evidence relating to the relationship between ZIKV and adverse neurological disorders during prenatal development [3]. An increase in the incidence rate of these neurological disorders has resulted in the World Health Organization (WHO) declaring ZIKV as a ‘public health emergency of international concern’. The WHO hopes to reduce the infection rate among pregnant woman by telling people to avoid travel to ZIKV-infected countries [4].

In recent years, vector-borne diseases such as dengue have become a major threat in Southeast Asian countries like India due to urbanization [5], the tropical climate, and poor waste management [6]. Messina et al. [6] conclude that people living in two million square kilometers of tropical and subtropical regions in the country are at high risk of contracting ZIKV. Although until now, there has been no transmission of ZIKA in India, it is important to consider where India is heading. Why has the red alert from the WHO made Indians more conscious of this virus even though it is has been known about for a long time? How we are going to combat this deadly disease until vaccines or antiviral drugs become available?

## Why India needs to be cautious?

In 1952, Smithburn et al. reported the serological evidence of ZIKV antibodies in India and described as a cross reactivity of other flaviviruses causing Dengue [7]. Oster et al. [2] reported sexual transmission of Zika in Dallas pushed the WHO to declare ZIKV a ‘public health emergency’, and warned African and Asian countries to take necessary precautions to avoid the entry of the virus, even though it causes deaths only rarely. A recent first report dated on Sep 30th 2016 on linking of Zika to birth defect with microcephaly in Thailand has insisted WHO to provide red-alert warning to Southeast Asian countries and take up precautionary measures in the spread of virus across Asia. Till date, 11 Southeast Asian countries namely Brunei, Myanmar, Cambodia, Indonesia, Laos, Malaysia, Maldives, Philippines, Thailand, Timor-Leste and Vietnam were reported with outbreak of Zika infection in occasional or smaller level. In addition to this, Singapore was added to travel notice list by CDC with the recent outbreak. In this context, India being a neighboring country, it is necessary to know the initiative step taken up by Indian government on awareness and spread of this disease. Although the Health Ministry of India has reported zero cases in India soon after a meeting held in Geneva by WHO on ZIKV, social activists and researchers are very much worried about under-reporting, misdiagnosis, or higher levels of immunity in places where the similar form of virus which causes dengue has been present for a longer time.

It is possible to find *A. aegypti* mosquitoes that transmit ZIKV wherever DENV is reported. *A. aegypti* and *A. albopictus* are the two commonest vectors responsible for transmitting DENV of four different strains in India: DENV-1, DENV-2, DENV-3, and DENV-4. A recent study by Musso and Gubler [8] concluded that the cocirculation of ZIKV with DENV and CHIKV might occur in countries where DENV and CHIKV are endemic. Data from the National Vector Borne Disease Control Programme state that 104935 cases of DENV (http://nvbdcp.gov.in/den-cd.html), and 57694 cases of CHIKV were recorded alone in 2016 (http://www.nvbdcp.gov.in/chik-cd.html) [9]. Among 104935 cases of DENV, 221 cases lead to death. The spread of the ZIKV is also alarming as it might lead to a potential increase in maternal mortality in India, where the rate is already high (15%compared to other nations) [10] and recorded AIDS-related maternal mortality was 2 080 in 2015.

India is having a trade relationship with most of the ZIKV affected countries in Latin America especially Mexico and Brazil. In Mexico, there are nearly 2 500 Indian diaspora members as Non Resident Indians in various firms such as businesspersons, scientists, researchers and students and reports also indicate that Mexico has become a route for illegal migrants from different countries including India. At the 7^th^ India-Brazil Joint Commission Meeting, one of the major points of discussion was boosting the trade relationship and establishing a forum of CEOs, giving way to the possibilities of frequent migration between the two nations. There are many Indian companies in Colombia associated with various industries, such as IT, pharmaceuticals, agrochemical, mining,etc. Recently Bogoch et al. [11] described India as the vulnerable country to Zika with 67 422 travellers arriving per year across the globe and 1.2 billion residents in India with potential Zika transmission areas. It is also worth noting that India is a country where prostitution is legal but restricted [12].

## Where India needs to concentrate? Exploring the possibilities

India is developing more trade relationships with ZIKA-affected countries where prostitution is legal, which can be an organized one for the route of Zika entry. This gives a clear indication that at least three ministries in the Indian context, such as the Ministry of Health, Ministry of Tourism, and Ministry of External Affairs, have to make efforts to curb the spreading of the ZIKV hand in hand.

National Centre for Disease Control (NCDC) was appointed as the nodal agency to monitor the outbreak of Zika in India. National Institute of Virology (NIV), Pune and NCDC, Delhi are the reference laboratories to investigate the outbreak and conform the laboratory diagnosis of Zika. Indian Council of Medical Research (ICMR) has recognised 10 National Virology laboratories across different states in India to test viral infections. Cases which were recorded for dengue and chikungunya as negative are being tested for Zika. In addition to this, Indian Ministry of Health has set up a ‘joint monitoring group’ under the Directorate General of Health Services (DGHS) and the ICMR to monitor the status of the ZIKV in India and advised women who are going to get pregnant or already pregnat to avoid travelling to Zika affected countries. This provides an additional prerequisite to the one already granted to pregnant women, especially by CDCs. Health ministry has been given proper instructions and advised women consult health practitioners before and after travelling to ZIKA-affected areas. Now, it is time for women to get extra attention when they are exposed to people returning from ZIKA-affected areas, especially during pregnancy.

In addition, the Health ministry has been giving proper instructions to women through various channels such as Ministry of Health and Family Welfare (MOHFW), Maternal and Child Health Division (under NHM) and Integrated Disease Surveillance Project (IDSP) to consult health practitioners before and after travelling to ZIKA-affected areas. In addition Rapid Response Teams (RRTs) comprising of epidemiologist/public health specialist, microbiologist and a medical/paediatric specialist and other experts (entomologist etc.) was initiated to monitor suspected outbreak. Billboards carrying information about Zika were displayed in various international airports and the travellers who are having febrile illness after visiting Zika affected areas are requested to report immigration authorities.

Nevertheless, the Indian government is also trying with the help of different agencies, such as the National CDC, the National Institute of Virology (NIV) in Pune, DGHS, IDSP well as specifically constituted rapid response teams, to eradicate the ZIKV. IDSP has provided national guidelines for Zika Virus Disease comprising of: Guidelines on Zika Virus Disease following Epidemic in Brazil and other countries of America, Guidelines for integrated vector Management for control of AEDES mosquito, Do’s and dont’s Zika Virus Disease, & Travel Advisory Zika Virus Disease and Zika Virus Fact Sheet (http://www.idsp.nic.in/index.php). In recent Union budget of 2015, Indian government has allocated 33 152 crores towards health sector. It has allocated 1214 crores by giving due importance to the ministry of Ayurveda, yoga and naturopathy, unani, siddha and homeopathy (AYUSH) for the promoting of traditional medicines (http://indiabudget.nic.in/budget.asp).

India has an approximate population of 1.32 billion with a sex ratio of 1.068, which is relatively high when compared to the global sex ratio. Out of this, 48.1% are females and infant mortality is around 30%. Having this stunning demographic data, representing over population prone to epidemic [13], it is evident that it is time for Indian government to allocate a considerable amount of funds towards the Zika campaign and other infrastructure facilities that supports testing and other progresses. Emergency allocation of fund is not impossible in India with 29 states having their own health budget apart from the union health budget. At the same time, the state governments have also strived to enhance their community programs to create awareness among people, especially among women. In Indian states where DENV is highly prevalent, citizens were asked to wear long sleeves and full pants to cover the entire body, utilize insecticide-treated bed nets, and use netted doors and windows in the home and permethrin-treated clothing and gear when outside to avoid getting mosquito bites. In addition to this, the best preventive measure for ZIKV will be the destruction of larval breeding sites, elimination of stagnant water, and the installation of proper sanitation facilities [6]. It is also advisable that the Indian Ministry of Electronics and Information Technology includes a ZIKV campaign in its Digital India website (http://digitalindia.gov.in/), which can reach the whole population, as digital media has its phases of development in drawing more attention for awareness perspective. A digital campaign on ZIKA is more evident in countries such as the US, where the CDC microsite is popular among several agencies. In addition to the above preventive measures, the NIV in Pune has been investigating ZIKV and has put forward an algorithm for diagnosing ZIKV in both infants and adults for early detection and treatment [6].

India is also one of the 14 potential developers for a vaccine against the ZIKV alongside nations such as the US, France, Austria, etc. Bharath Biotech from India is in the race of developing two candidate vaccines (recombinant and inactivated) towards ZIKA [14]. Though it is encouraging that Bharath Biotech recently claimed that it had identified two variants, which are ready for preclinical studies, to which extent this is going to be put into practice is a question among many. Reports also state that it will take nearly 18 months to conclude the vaccine variant trial. Nevertheless, researchers are also attempting to develop a biopesticide to kill mosquito larvae and adult mosquitoes [15], a long-acting mosquito repellent [16], and a mosquito trap surveillance system. Gyawali et al. [17] describe effective methods to combat the ZIKV such as practicing safe sex, implementing safety measures in blood and blood-related ZIKV transmission targets, raising public awareness, developing vaccines, implementing vector control programs, and also by public and private sectors prioritizing research on the ZIKV. In addition, it is mandatory for one to be careful during sex of any kind (vaginal, oral& anal) by using condoms, dental dams, or avoiding sex during the time of the entire pregnancy. This will ensure an effective surveillance approach for pregnant women who are returning from endemic regions.

## Conclusions

Though there is no evidence of ZIKV transmission in India, recent reports from neighbouring Southeast Asian countries such as Singapore, Indonesia and Thailand of Zika transmission raise many queries that remain unanswered to this date. A recent report suggested second largely populated country India is vulnerable to Zika has created a need for a discussion on how awareness will be raised and what preventive measures will be taken in the Asian perspective. It is also considered to be an indigenous factor that the term Zika was discussed long ago, alarming at the end of all the corners of India on dengue, making Indian researchers more cautious when determining the route cause and applying proper research to identify and curb the deadly Zika disease. Dengue in the Indian perspective has its own dimension with the prevalence of different serotypes in different tropical conditions. However, it is not so in other countries where one or two serotypes has its own feet. Taking all into consideration, continued surveillance and effective preventive measures will be the first step to stop the Zika invasion in newly industrialized India. Nevertheless, the Indian government had also taken enough measures through Rapid Response Teams in monitoring the Zika outbreak, preventive measures as well as to develop mutiple vaccines against ZIKV. Moreover, it is the right time for India to launch a massive campaign against Zika including in the agenda “Clean India” on a priority basis.

## Additional file

Additional file 1: Multilingual abstracts in the five official working languages of the United Nations. (PDF 512 kb)

## Abbreviations

AYUSH: Department of Ayurveda, Yoga, and Naturopathy, Unani, Siddha, and Homoeopathy
CDC: Center for Disease Control and Prevention
CHIKV: Chikungunya virus
DENV: Dengue virus
DGHS: Directorate General of Health Services
DGHS: Director General of Health Services
IDSP: Integrated disease surveillance programme
NIV: National Institute of Virology
NVBDCP: National Vector Borne Disease Control Programme
RRTs: Rapid response teams
WHO: World Health Organization
ZIKV: Zika virus
