# Supplementary material for: Zika: How safe is India?
Source: Infect Dis Poverty. 2017 Jan 31;6:37. doi: 10.1186/s40249-016-0234-6 (PMC5295178; doi:10.1186/s40249-016-0234-6)
Supplement: Additional file 1: — Multilingual abstracts in the five official working languages of the United Nations. (PDF 512 kb) [file 40249_2016_234_MOESM1_ESM.pdf]

Translation of the abstract into the six official working languages of the United Nations

زيكا: هل الهند آمنة؟

جورج بريا دوس س، سيفا ر، برابو كريستوفر ب، تشيرانجيب تشاكرابورتي، هايلونق زو

خلاصة:
فيروس زيكا، والذي نشأ من غابة في أوغاندا، قد أصاب عدة دول في أفريقيا، أمريكا اللاتينية وآسيا. أغلب الأشخاص المصابين بالفيروس خالين من الأعراض ويظهرون أعراضا مرضية متراوحة من ارتفاع طفيف في درجة الحرارة إلى اضطرابات عصبية شديدة. ومع انتشار العدوى مؤخرا إلى البلدان في جنوب-شرق آسيا، فقد حذر مركز *مكافحة الأمراض واتقائها* النساء الحوامل من زيارة 11 دولة آسيوية إلا للضرورة. ظهور بعض التقارير عن إمكانية نقل الفيروس عن طريق الالتقاء الجنسي حث *منظمة الصحة العالمية* على إعلان أن فيروس زيكا "خطرا يهدد الصحة العامة". وبذلك أصبح من اللازم معرفة موقف دولة الهند، الثانية عالميا في الازدحام السكاني، بهذا الشأن من ناحية نشر الوعي واتخاذ اجراءات احتياطية ضد عدوى فيروس زيكا. هدف هذه الدراسة إلقاء الضوء على أهمية زيكا في المجتمع الهندي من خلال عدة مؤشرات مثل الحجم السكاني والنسب السكانية، معدل الوفيات، الأمراض المرتبطة، المبادرات الحكومية، وغيرها من العوامل على المستوى الدقيق والمعرضة لتأثير زيكا.

Translated from English version into Arabic by SAlkhodair, through

TRANSLATORS
WITHOUT BORDERS

寨卡: 印度有多安全?

George Priya Doss. C, Siva R, Prabhu Christopher B, Chiranjib Chakraborty, Hailong Zhu

摘要:
源于乌干达森林的寨卡病毒，至今已蔓延至非洲，拉丁美洲及亚洲的多个国家。大部分寨卡病毒感染并没有明显的病症，其临床表现可为轻微的发烧至严重的神经性疾病。最近，由于寨卡病毒在东南亚地区的爆发，美国疾病控制与预防中心已针对 11 个亚洲国家发出预警，敦促人们，特别是孕妇，若非必要应避免到这些国家旅行。此外，世界卫生组织将寨卡病毒传播宣布为“突发公共卫生事件”。因此，作为一个临近寨卡感染地区的世界人口大国，印度必然需要检视关于寨卡病毒传播的公众意识及相应的预防措施的现状。本文旨在反映印度在应对寨卡病毒方面的现状及所面临的重要工作，阐述了相关的一些宏观指标，如人口基数及比率、死亡率、相关疾病、政府举措，以及其他影响寨卡病毒传播的若干微观因素。

Translated from English version into Chinese by Hailong Zhu

Zika: l’Inde est-elle protégée?

George Priya Doss. C, Siva R, Prabhu Christopher B, Chiranjib Chakraborty, Hailong Zhu

Résumé
Le virus Zika, qui provient d’une forêt ougandaise, a affecté des pays d’Afrique, d’Amérique Latine et d’Asie. Les personnes infectées sont, pour la plupart, asymptomatiques, et présentent des manifestations cliniques allant d’une fièvre légère à des troubles neurologiques graves. En raison des récentes épidémies survenues dans les pays du Sud-Est asiatique, le Centre de Contrôle et de Prévention des Maladie a mis en garde les femmes enceintes afin qu’elles évitent les voyages non essentiels dans 11 pays d’Asie. Des rapports sur les voies de transmission sexuelle du Zika ont incité l’Organisation Mondiale de la Santé à déclarer ceci comme une «urgence de santé publique». Dans l’état actuel de mise en garde, il devient obligatoire de prendre en considération la place de l’Inde, deuxième pays le plus peuplé du monde, concernant la sensibilisation et la prise de mesures de précaution contre l’infection par le virus Zika. Par conséquent, cet article a pour objet de souligner l’importance du virus Zika dans la population indienne, en prenant en compte plusieurs indicateurs, tels que la densité et les ratios de la population, le taux de mortalité, les maladies étroitement liées, les initiatives gouvernementales, ainsi que d’autres micro-facteurs exposés aux effets du virus Zika.

Translated from English version into French by Ode Laforge, through

TRANSLATORS
WITHOUT BORDERS

Вирус Зика: насколько это безопасно в Индии?

Джордж Прийя Досс. С., Сива Р., Прабху Кристофер Б., Чиранджип Чакраборти, Хайлонг Чжу
George Priya Doss. C, Siva R, Prabhu Christopher B, Chiranjib Chakraborty, Hailong Zhu

Реферат

Возникший в лесу Уганды вирус Зика затронул страны Африки, Латинской Америки и Азии. У большинства инфицированных этим вирусом болезнь протекает бессимптомно и сопровождается широким спектром клинических проявлений: от незначительной лихорадки до серьезных неврологических нарушений. В связи с недавними вспышками болезни в странах Юго-Восточной Азии Центры по контролю и профилактике заболеваний США предупредили беременных женщин, чтобы они избегали несущественных поездок в 11 азиатских стран. Сообщения о передаче вируса Зика половым путем заставили Всемирную организацию здравоохранения объявить вирусную инфекцию Зика «чрезвычайной ситуацией в области общественного здравоохранения». Учитывая нынешний статус предупреждения, необходимо рассмотреть, в каком состоянии находится повышение информированности населения и принятие мер предосторожности против вирусной инфекции Зика в Индии, второй по численности населения стране. Поэтому в данной работе, цель которой – подчеркнуть значение вируса Зика для индийского населения – рассматриваются некоторые индикаторы, такие как численность и соотношение населения, показатели смертности, близкородственные заболевания, инициативы правительства, а также другие микроуровневые факторы, подверженные воздействию вируса Зика.

Translated from English version into Russian by Tatyana Johnson, through

TRANSLATORS
WITHOUT BORDERS

Zika: ¿Qué tan segura es India?

George Priya Doss. C, Siva R, Prabhu Christopher B, Chiranjib Chakraborty, Hailong Zhu

Resumen

El virus del Zika, originario de un bosque en Uganda, ha afectado a países en África, América Latina y Asia. La mayoría de las personas infectadas por el Zika es asintomática y presenta manifestaciones clínicas que varían entre una leve fiebre y severos trastornos neurológicos. Ante los recientes brotes en países del Sudeste Asiático, los Centros para el Control y Prevención de Enfermedades han recomendado que las mujeres embarazadas eviten viajes innecesarios a 11 países de Asia. Los informes sobre la vía sexual de transmisión del Zika han llevado a la Organización Mundial de la Salud a declarar una “emergencia de salud pública”. Con el presente estado de advertencia, ha pasado a ser una obligación evaluar en qué situación se encuentra India, el segundo país más poblado, en cuanto a la difusión de la concientización y la toma de medidas de precaución contra la infección por el virus del Zika. Por lo tanto, este artículo tiene como objetivo resaltar la importancia del Zika en la población de la India mediante la evaluación de varios indicadores, como tamaño y proporción de la población, índices de mortalidad, enfermedades altamente relacionadas, iniciativas gubernamentales y otros factores a un nivel más específico que son propensos a los efectos del Zika.

Translated from English version into Spanish by Maria Alejandra Aguada, through

TRANSLATORS
WITHOUT BORDERS
